# Charlevoix-Saguenay spastic ataxia: a novel mutation in the SACS gene

**DOI:** 10.1007/s13760-025-02813-z

**Published:** 2025-06-05

**Authors:** Lan Li, Xiuhua Tian, Junfeng Chen, Xiaoyan Guo

**Affiliations:** Department of Neurology, The Affliated Hospital of Southwest Medical University, No. 25 of Taiping Street, Luzhou, 646000 Sichuan China

**Keywords:** Autosomal recessive ataxia of CharlevoixSaguenay, SACS gene mutation, Frameshift mutation, Ataxia

## Introduction

Autosomal recessive spastic ataxia of Charlevoix-Saguenay (ARSACS) is a complex disorder caused by mutations in the SACS gene located on chromosome 13q12.12. It was initially identified in French Canada, in the Charlevoix and Saguenay-Lac-Saint-Jean regions of Quebec. The clinical phenotypes include early onset cerebellar ataxia, axonal-demyelinating sensorimotor peripheral neuropathy, and pyramidal tract signs. Slowly progressive ataxia is accompanied by limb spasms due to progressive degeneration of the corticospinal and spinal cerebellar tracts [1]. Most patients exhibit typical peripapillary retinal streaks on ophthalmoscopy and thickened peripheral retinal nerve fiber layers on optical coherence tomography (mean > 119 μm) [2]. Brain magnetic resonance imaging (MRI) revealed cerebellar atrophy and hypointensity of the pons on T2-weighted and fluid-attenuated inversion recovery (FLAIR) axial sequences. Electrophysiological studies showed mixed demyelination and axonal neuropathy [3]. Recent studies have shown that ARSACS is not limited to Quebec, Canada. It exists in various forms worldwide [3]. Here, we describe the clinical and molecular characterization of a 31-year-old Chinese male with a novel nonsense and frameshift SACS mutation.

## Case presentation

A 31-year-old man presented with a history of gait problems that began in childhood. Compared to his peers, he was prone to falls while running and experienced leg stiffness during walking. At 27 years of age, he developed significant weakness in both lower limbs. The patient was born to a non-consanguineous family. The patient’s sister has similar gait problems. At the age of 10, she began to have difficulty walking and had frequent falls, which gradually worsened over time, necessitating the use of a wheelchair.

On neurological examination, the patient presented with mild cerebellar dysarthria, which manifests as slowed and slurred speech, but without dysphagia. Distal weakness was present in the lower limbs. Dysarthria, bilateral horizontal gaze-induced nystagmus, and pes cavus were also noted. Deep tendon reflexes were brisk in the knees and absent in the ankles. Superficial and deep sensations in the distal extremities were mildly reduced. Extensor plantar responses, cardiac abnormalities, hearing loss, vision problems, autonomic dysfunction, and seizures were also observed.

**Fig. 1:**
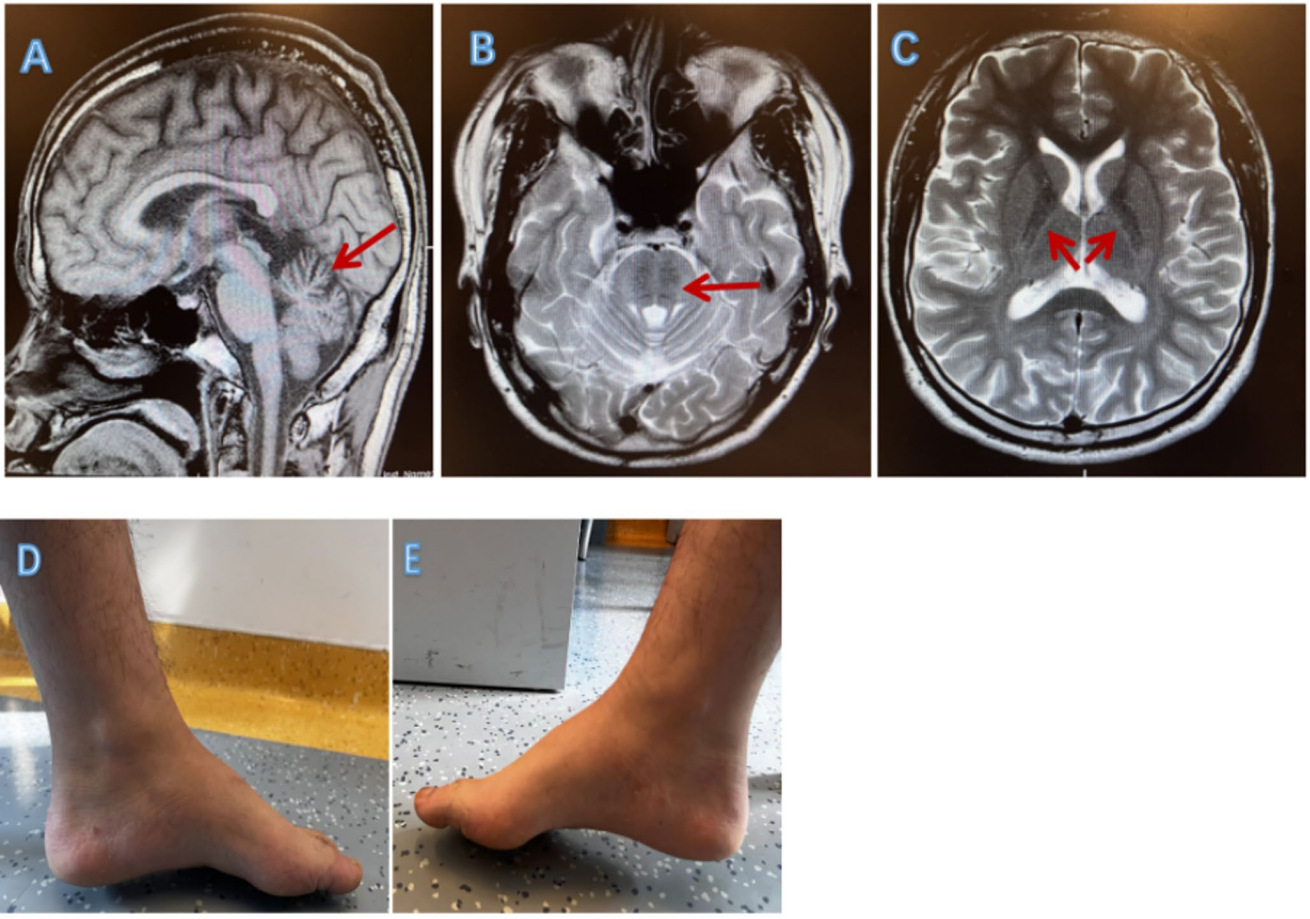
Clinical and neuroimaging characteristics of the patient. (**A**) Atrophy of the cerebellar vermis on the sagittal T2-weighted image (red arrows); (**B**) Cross-sectional T2-weighted image showing pontine hypointensity (red arrows); (**C**) T2 hyperintense margins around the thalamus on either side of the cross-section (red arrows); (**D–E**) pes cavus and hammertoes in patient

Routine investigations—including complete blood counts (white blood cell count, white blood cell differential, red blood cell count, platelet count, hemoglobin measurement, etc.), serum creatinine and electrolytes, serum transaminase levels, stool and urine, coagulation tests, autoantibody profiles, a complete set of thyroid functions, and cerebrospinal fluid biochemistry—were all within normal limits. The patient’s fundus appeared normal, with no signs of retinal nerve fiber layer thickening. Nerve conduction studies show that the latent period of motor and sensory conduction terminals of the bilateral tibial nerve and common peroneal nerve is within the normal range, and the conduction velocity is slowed and the amplitude is reduced. The brain MRI showed cerebellar atrophy and linear hypointensities in T2 and T2-FLAIR images in the pons. Interestingly, a T2 high signal rim around the thalamus on both sides were also observed (Fig. 1).

**Fig. 2:**
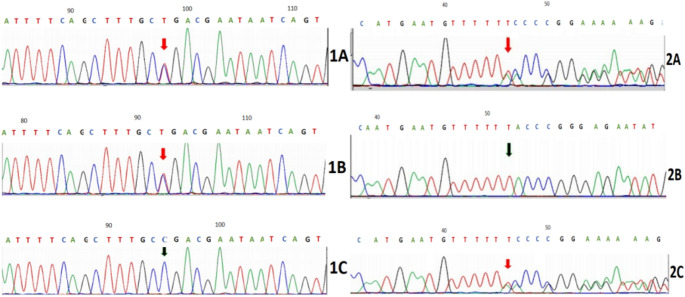
Identification of mutations of the SACS gene.The sequences in the patient (1 **A**; 2 **A**), father (1 **B**; 2 **B**), and mother(1 **C**;2 **C**) are shown

We screened for the causative genes of SCA 2 and 3 but did not find any abnormal amplification of SCA causative gene repeat sequences. Finally, the patient underwent whole-exome analysis, and heterozygous mutations in *SACS* were detected, which were compound heterozygous nonsense mutations and frameshift mutations c.7273 C > T (p.Arg2425) and c.12529 del (p.Tyr4177 Thrfs*45). Among them, the c.7273 C > T mutation was evaluated as pathogenic according to the American Society of Medical Genetics and Genomics’ variant interpretation guidelines. The c.7273 C > T mutation was found in the unaffected father and c.12529 del in the unaffected mother (Fig. 2). During the 3, 6, and 12-month follow-up calls, his condition remained stable.

## Discussion

Charlevoix-Saguenay autosomal recessive spastic ataxia (ARSACS) is the second most common autosomal recessive ataxia after Friedrich ataxia and was diagnosed in 1978 among French Canadian patients in the Charlevoix-Saguenay-Lac-Saint-Jean (Lac) region of Quebec, Canada. The *SACS* gene is located on chromosome 13q12 and consists of 10 exons, 9 of which are coding exons [1]. Sacsin is an ataxic protein that is most abundantly expressed in the central nervous system, particularly in the cerebral motor system, and in the Purkinje cells of the cerebellum [4]. It interferes with the Hsp70 molecular chaperone machinery, reducing the involvement of this molecular chaperone in the processing of other ataxia-related proteins, which in turn leads to neurodegenerative consequences [5]. SACS usually presents in childhood. The latest reported age of onset for ARSACS was 60 years, as described by Radziwonik et al. [6]. Cerebellar ataxia, lower limb spasticity, and peripheral neuropathy are the classic triad of ARSACS, and common clinical features include dysarthria, nystagmus, finger and foot deformities, and retinal myelodysplasia. urinary dysfunction, among others [1, 3].

Here, we report a new case of SACS gene mutations, c.7273 C > T and c.12529 del, characterized by lower-limb spasticity, peripheral neuropathy, cerebellar atrophy, and pontine linear hypointensities. However, our patient did not exhibit significant ataxia, which is a typical clinical feature of ARSACS. Our patients had mild dysarthria and horizontal-gaze nystagmus along with cerebellar atrophy on MRI, but no gait, trunk, or limb ataxia, suggesting some clinical variability in SACS. We consider that this may be related to the site of the *SACS* gene mutation. The clinical presentation in this case is caused by a compound heterozygous mutation in the *SACS* gene.The c.7273 C > T site mutation leads to premature termination of amino acid translation of the encoded protein, and the protein is truncated short. The gnomAD database revealed that the allele frequency of this variant in the total population was < 0.01% (3/249774). The c.12529 del mutation is located in the last exon of the transcript NM_014363.5, which results in a frameshift mutation in the amino acid of the encoded protein (tyrosine mutation to threonine); translation is terminated after the frameshift mutation of the amino acid. This novel mutation is not yet included in the HGMD database. We believe that the slow progression of the patient’s symptoms may be related to frameshift mutations, and a growing number of studies have begun to report atypical cases that do not necessarily present with classic ataxia and spasticity but may present with cognitive and psychiatric symptoms and epilepsy [7–10]. The phenotype of this patient was milder than that of a typical ARSACS patient, and he carried heterozygous mutations in two *SACS* genes, one of which was a newly discovered c.12529 del mutation, which may be an important cause of the patient’s mild phenotype. In addition, the patient presented with other neurological symptoms, such as spasticity and mild peripheral neuropathy, in the absence of typical ataxic manifestations, further emphasizing the clinical heterogeneity of ARSACS. Further study of these atypical manifestations will help us understand the diversity of *SACS* gene mutations and their effects on different regions of the nervous system. The UBD domain mediates protein-protein interactions by binding ubiquitin or ubiquitin-like modifications. Similarly, a previously reported patient with homozygous variants downstream of the UBD domain (c.11,104 A > G) presented with non-ataxia spastic paraplegia [10]. Truong et al. reported two patients presenting with peripheral neuropathy without ataxia and spasticity, who were eventually diagnosed with CMT and harbored mutations in the *SACS* gene [11]. Studies have shown that there is some overlap in the clinical manifestations of ARSACS and hereditary peripheral neuropathy, especially in some atypical cases. Therefore, neuroimaging is needed for differential diagnosis in clinical practice [12]. In this case, the patient had highly specific linear pontine T2 hypointensity and T2 hyperintense margins around the bilateral thalamus, which may serve as diagnostic markers for imaging. Therefore, when the clinical presentation is atypical, it is necessary to complete SACS genetic testing to confirm the diagnosis.

## Conclusion

This paper reports a de novo mutation in the *SACS* gene (c.12529 del) that helps assess the association between genotype and phenotype.

## Supplementary Information

Below is the link to the electronic supplementary material.

Supplementary material 1 (DOCX 17.6 kb)

## Data Availability

No datasets were generated or analysed during the current study.
