# Supplementary material for: Charlevoix-Saguenay spastic ataxia: a novel mutation in the SACS gene
Source: Acta Neurol Belg. 2025 Jun 5;125(5):1421–4. doi: 10.1007/s13760-025-02813-z (PMC12518459; doi:10.1007/s13760-025-02813-z)
Supplement: Supplementary file 1 — Supplementary material 1 (DOCX 17.6 kb) [file 13760_2025_2813_MOESM1_ESM.docx]

| literature | Mutation sites | Main manifestations | Electrophysiological studies |
| --- | --- | --- | --- |
| Liu L,et al^.[1]^（2016）China | c.11803C>T；Chromosomal deletion | peripheral neuropathy，spastic gait | Electrophysiological studies revealedperipheral neuropathy with mixed axonal degeneration and demyelination findings. The motor nerve conduction velocities (MNCVs) were  moderately reduced and the  amplitudes of compound muscle action potentials (CMAPs) were decreased. The sensory nerve conduction velocities (SNCVs) were reduced and the amplitudes of sensory nerve action potentials (SNAPs) were  decreased |
| Sahib A,et al.^[2]^（2024）India | c.5151del；c.9175A>T | gait ataxia | Nerve conduction study revealed mixed sensorimotor, predominantly demyelinating polyneuropathy |
| Sugumaran R,et al.^[3]^ （2024）India | c.11938G>A ；chr13:g.23,906,077 C>T | Walking unsteadily | Nerve conduction study (NCS) showed severe sensorymotor predominantly demyelinating peripheral neuropathy  with secondary axonal changes |
| Aida I,et al.^[4]^（2021）Japan | c.12976A>G；c.4233-4236  delACTT | gait disturbance ； cerebellar ataxia | In nerve conduction studies, in the ulnar and median nerves, the amplitudes of CMAP were moderately reduced and the motor  nerve conduction velocities were slightly decreased |
| Cho H,et al.^[5]^（2021）Korean | c.7272C>A；c.11319_11321del | gait difficulty；dysarthria ； dysphagia | Neurophysiologic studies were consistent with predominantly demyelinating sensori-motor polyneuropathy |
| Ricca I,et al.^[6]^ （2019）Italy | P1:c.563G>A；c.7394C>T；c.11747T>G  P2：c.962G>A；c.8330G>A | P1:spastic-ataxic gait； mild dysarthria  P2:ataxia | P1:severe sensorimotor axonal and demyelinating  Polyneuropathy  P2:Neurophysiological examination was consistent with  sensorimotor axonal polyneuropathy |
| Saffie P,et al.^[7]^ (2017) Chile | c.4492C.T ;c.2388dupA | spastic ataxia,hyperreflexia, pes cavus, | axonal polyneuropathy |
| Palmio J,et al.^[8]^(2016) Finland | c.3298G>A;4466A>G；c.4076T>C （The mutation sites were the same in both patients） | P1:muscle weakness;spasticity;ataxia  P2:ataxic gait | P1:NCVs were decreased  P2:Electrophysiological studies showed decreased sensory and motor NCVs |
| Mignarri A,et al.^[9]^（2014）Italy | c.1373C>T;exon 10 duplication | gait unsteadiness;psychiatric symptoms | Nerve conduction study uncovered axonal sensory neuropathy |
| Masciullo M,et al.^[10]^(2012) Italy | P1:c.11598delC;c.4108C>T  P2:c.11777A>G;c.11265_11266delAT | P1:spasticity LL,  Brisk reflexes LL,  bilateral ankle clonus  and Babinski sign  P2:LL spasticity  brisk reflex LL and Babinski sign | NCS: A-D  polyneuropathy |
| Tzoulis C,et al.^[11]^(2013) Norway | c.13352T>C;c.6890T>G | LLstiffness,gait unsteadiness,dysarthria, dysphagia | Electromyography (EMG) and nerve conduction velocity (NCV) studies were consistent with a predominantly axonal sensorimotor peripheral neuropathy. |

1. Liu L, Li XB, Zi XH, et al. A novel hemizygous SACS mutation identified by whole exome sequencing and SNP array analysis in a Chinese ARSACS patient. J Neurol Sci. 2016;362:111-114.
2. Sahib A, Choudhury C, Nagar R, Koul A. Autosomal Recessive Spastic Ataxia of Charlevoix-Saguenay Secondary to a Novel Mutation in the SACS Gene. Ann Indian Acad Neurol. 2024;27(5):586-587.
3. Sugumaran R, Bhuvaneswaran R, Narayan SK. Autosomal recessive spastic ataxia of charlevoix-saguenay (ARSACS)-first with tongue wasting, peripheral nerve thickening and a novel SACS gene mutation. Acta Neurol Belg. 2024;124(6):2039-2042.
4. Aida I, Ozawa T, Fujinaka H, Goto K, Ohta K, Nakajima T. Autosomal Recessive Spastic Ataxia of Charlevoix-Saguenay without Spasticity. Intern Med. 2021;60(24):3963-3967.
5. Cho H, Lyoo CH, Park SE, Seo Y, Han SH, Han J. Optical Coherence Tomography Findings Facilitate the Diagnosis of Autosomal Recessive Spastic Ataxia of Charlevoix-Saguenay. Korean J Ophthalmol. 2021;35(4):330-331.
6. Ricca I, Morani F, Bacci GM, et al. Clinical and molecular studies in two new cases of ARSACS. Neurogenetics. 2019;20(1):45-49.
7. Saffie P, Kauffman MA, Fernandez JM, Acosta I, Espay AJ, de la Cerda A. Teaching Video NeuroImages: Spastic ataxia syndrome: The Friedreich-like phenotype of ARSACS. Neurology. 2017;89(14):e178-e179.
8. Palmio J, Kärppä M, Baumann P, Penttilä S, Moilanen J, Udd B. Novel compound heterozygous mutation in SACS gene leads to a milder autosomal recessive spastic ataxia of Charlevoix-Saguenay, ARSACS, in a Finnish family. Clin Case Rep. 2016;4(12):1151-1156. Published 2016 Oct 26.
9. Mignarri A, Tessa A, Carluccio MA, et al. Cerebellum and neuropsychiatric disorders: insights from ARSACS. Neurol Sci. 2014;35(1):95-97.
10. Masciullo M, Modoni A, Tessa A, et al. Novel SACS mutations in two unrelated Italian patients with spastic ataxia: clinico-diagnostic characterization and results of serial brain MRI studies. Eur J Neurol. 2012;19(8):e77-e78.
11. Tzoulis C, Johansson S, Haukanes BI, Boman H, Knappskog PM, Bindoff LA. Novel SACS mutations identified by whole exome sequencing in a norwegian family with autosomal recessive spastic ataxia of Charlevoix-Saguenay. PLoS One. 2013;8(6):e66145. Published 2013 Jun 13.
